# Risk equivalence as an alternative to balancing mean value when trading draft selections and players in major sporting leagues

**DOI:** 10.1371/journal.pone.0217151

**Published:** 2019-05-24

**Authors:** Geoffrey N. Tuck, Shane A. Richards

**Affiliations:** 1 Commonwealth Scientific and Industrial Research Organisation Data 61, and Oceans and Atmosphere, CSIRO Marine Laboratories, Castray Esplanade, Hobart, Tasmania, Australia; 2 School of Natural Sciences, University of Tasmania, Hobart, Tasmania, Australia; Universitat Jaume I, SPAIN

## Abstract

In sports leagues that use an annual draft to assign eligible players to clubs, having a value associated with a draft selection can allow clubs to anticipate future growth of players and, if a trading period exists, assist negotiations when exchanging draft selections and players. Typically, mean draft values often decline in either an exponential or geometric manner with increasing draft selection number. Aggregate mean values have been used to compare trade packages. However, clubs may also want to ensure that a trade does not increase the probability of obtaining poor players in the draft. This paper therefore considers equivalence of risk as an alternative trading strategy for club list managers. Here, risk is defined as the probability of the aggregate value of the received draft selections being below a minimum acceptable level. For risk equivalence, a premium over and above mean market value may need to be provided when trading to secure higher draft selections.

## Introduction

Valuing a player’s potential as an individual and as part of the greater team structure is a critical component of managing any major sporting club [1–5]. In many sporting leagues, an annual reverse-order draft (or variant) is used to distribute eligible players to the league’s clubs. In a reverse-order draft, clubs select players from the draft pool in-turn, from the poorest performing to the best performing club. One purpose of the reverse-order draft is to encourage competitive balance [5–8]. In leagues that allow selections and players to be traded between clubs prior to the draft, having a “fair value” [9] for a particular draft selection (or selections) relative to others can facilitate trade negotiations [10, 11, 12]. Many studies have developed methods to value player performance, and others have also related this to the position players are drafted [1, 12–17].

**Fig 1:**
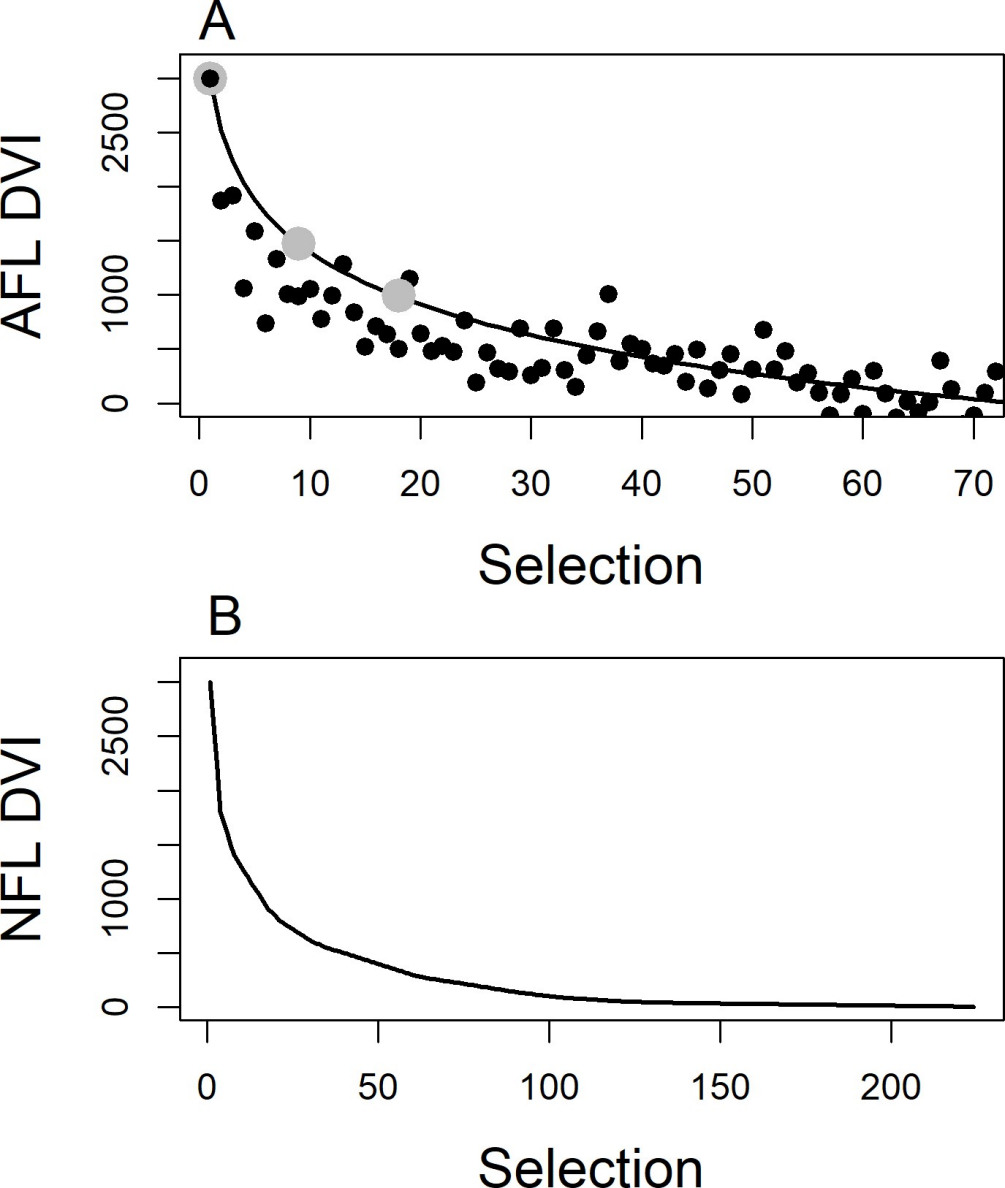
Draft Value Indices (DVI) by selection number. (A) The DVI (solid line) used by the Australian Football League (re-scaled to 3000 at selection number 1). Solid black points are salary data from which the DVI is derived. Solid grey points are draft values at selections 1, 9 and 18. (B) The DVI for the National Football League (NFL).

Functions defining value by draft selection number, here referred to as Draft Value Indices (DVI) but also known as pick value charts or value functions, have been estimated using various metrics across a number of sports (Fig 1) [9, 11, 12, 18]. The Australian Football League (AFL) recently introduced a DVI to assist clubs value their Academy and Father-Son players in the draft. AFL Club Academies were introduced in 2009 to allow clubs in traditionally non-AFL Australian states to preferentially select young talented players from those states, and father-son selections allow the club of a past player priority selection of the player’s son in the National Draft. In the bidding process for players, Academy and Father-son players acquire a 20% discount in point value relative to the value required by another club bidding for the same player. The AFL DVI is based upon historical average salaries by draft selection (Fig 1A) [9]. In the National Football League (NFL), the Chart (Fig 1B), as it is commonly known, shows the typical exponential decline from high (the initial selections, with the first selection having a re-scaled value of 3000 points) to low draft selections. The Chart was estimated in 1991 by staff of the Dallas Cowboys using trades from 1987 to 1990 [12, 19]. Massey and Thaler [12] compare the ratio of market values of alternative draft selections (from the Chart) against surplus value (defined as the performance value less the salary paid). They find that draft value mimics the Chart well, most likely because clubs are using the Chart in their trade negotiations (it has become the norm by which trades are valued). However, they also find that high draft selections in the Chart are over-valued when compared to lower round draft selections, in particular with regard to their value-for-money, and clubs holding a high draft selection should therefore trade down (obtain lower draft selections). They state a number of potential reasons for the apparent greater value placed on high draft selections, largely based on behavioural psychology. They conclude that the market for draft selections in the NFL is an example of an inefficient equilibrium and the Chart is “wrong”. In the Discussion of this paper, a further view is provided, namely that the structural form of the Chart may actually be a consequence of club recruiting staff having intuitively balanced risk in their trades over the 1987 to 1990 trading period.

Recruiting players to clubs is inherently risky. Clubs invest substantial financial resources to manage their player lists. This includes determining players to retain, de-list, trade and recruit through the draft. There is considerable risk that players drafted or traded-in will not meet expectations, and clubs minimise this risk through extensive background research, including tests of character, skill, competitiveness, leadership and injury history [5, 11]. However, there remains a substantial likelihood that a player will not fulfil their own expectations or those of their club. The expectation of club members and supporters, along with substantial media scrutiny, places additional pressure on clubs for a successful outcome in trading (of draft selections or players) and recruiting of players in the draft [20, 21].

The decision to trade players or draft selections (or a combination thereof) to another club often leads to protracted negotiations where each club attempts to maximise their potential gains (or perhaps minimise their losses). Such trade negotiations should, ideally, lead to a fair exchange (as implied in the examples provided within [9]), but will be constrained by the players and selections available to be traded [11]. In such circumstances, measures of draft selection value or player value can be used to assist negotiations [1, 11, 12]. Draft Value Indices have been used to help balance trades in the NFL and more recently in the AFL [9, 12]. The trades, and the DVI upon which they are based, implicitly assume that the exchanges will provide equivalence in mean performance (and in aggregate, if more than one selection is traded) [9]. Whether performance of the recruited players meets expectation in reality is purely probabilistic and, as such, a closer consideration of the distribution (and in particular the variance) of possible outcomes can be enlightening. As illustrated by Schuckers [11] and Hurley *et al* [19], distributions of performance are highly skewed with a high density of zeroes, depending on the metric chosen, for lower draft selections. This immediately leads to questions of whether the measure of central tendency used to compare trades should be the mean or instead the median. The median is generally considered the better measure of central tendency when data are skewed [22]. Furthermore, is central tendency the appropriate currency when negotiating or comparing trades when the risk of obtaining a poor player is very large? While the measure of central tendency may be equivalent in a trade, the probability of obtaining players of satisfactory productivity may not be.

There are many ways to define risk, but here risk is defined as the probability of the aggregate value of the received draft selections being below a minimum acceptable value (MAV). Using this as the basis for trade negotiations, clubs will then want to ensure that aggregate draft value is above this level with sufficient probability. As with the measure of central tendency, defining a risk probability is subjective, but for illustrative purposes it is here set to α = 0.05. The player selection value corresponding to this is the minimum accepted value. This implies that there is a probability of 0.05 of receiving a worse combination of selections than the MAV (and therefore a probability of 0.95 of obtaining a better selection than the MAV). Alternative risk probabilities could be chosen, depending on the level of risk-aversion of recruiting staff, with α = 0.05 being consistent with common tests of statistical significance. The MAV associated with a trade is defined by the following distribution function:

$$F_{T_{j}}({MAV}_{T_{j}})=Pr\left(\sum_{i\in T_{j}}{DVI}_{i}\leq {MAV}_{T_{j}}\right)=\alpha$$

where *T*_*j*_ is the set of selections associated with the trade involving club *j*, DVI_*i*_ is the DVI for selection *i*, and α is the risk (here set at 0.05). Thus, for a specified risk, α, trade *T*_1_ is deemed better than trade *T*_2_ if MAV_*T*1_ > MAV_*T*2_.

Strategies that attempt to balance risk across alternative management options are also known in gambling, finance, medicine and natural resource management. In finance, for example, in order to encourage investment in a risky asset compared to a risk-free asset with the same mean return, an additional risk premium over and above the mean return may be required [23]. In medicine, treatments may vary in performance; with one treatment having a greater mean increase in patient longevity but also greater risk of failure compared to another treatment that is more certain in its effect but has a lower mean resulting longevity [24]. In this case, the risk premium is the amount of an additional incentive (e.g. financial or a supplementary treatment) required for the patient to choose the initially riskier treatment. In fisheries management, where alternative methods for estimating fish abundance (and the consequent allowable catch quota) exist that vary in implementation cost and precision, a risk premium in the form of reduced catch may be required if managers choose to use cheaper and less precise (and therefore riskier) estimation methods [25, 26, 27].

Recall that risk is defined as the probability of the aggregate value of the received draft selections being below a minimum acceptable value. Therefore, for risk equivalency, clubs will seek trades that balance the probability of receiving selections of a minimum (aggregate) draft value. For example, if a club holds a draft selection (or selections) with a 0.05 probability of being less than the MAV, then trade negotiations with another club will minimally need to meet this requirement. Clubs can achieve this by packaging selections (or players if these have been valued using a similar acceptable metric). However, to determine the MAV given a risk probability requires more than the DVI (mean market values) that have been used in the NFL and AFL to date, where the mean DVI for each selection can be added to obtain an aggregate total value for the selection package [5, 9, 12]. With risk equivalency, the full probability density distribution of value is needed and risk value percentiles with such skewed distributions are not additive.

This paper illustrates the concept of risk equivalence in trade negotiations for draft selections and proposes a mechanism for determining fair trades based upon risk rather than, or in addition to, mean market value. The motivation for this results from the realisation that while average player value may be maintained during an exchange of draft selections or players, the risk taken by one of the clubs may be significantly greater than its counterpart. Importantly, this research may influence how recruiting staff and list managers approach trading. The paper first provides a simple example to illustrate the concept of risk equivalency, and then moves to more pragmatic examples using data from the AFL, where past trades in the AFL are evaluated for their risk and mean value equivalency.

## Methods

### An illustrative hypothetical example

To illustrate the alternative strategies of balancing the mean DVI value or balancing risk for trading draft selections, an example is provided where comparisons are made between outcomes from a single draft selection (selection 1 in this case) and multiple lower draft selections. Mean values from the AFL DVI are used, where the DVI (as a function of selection number) has been re-scaled, following the NFL and AFL, so that the number 1 selection has a point value of 3000 (Fig 1A). The AFL DVI is based upon average salary information at each draft selection [9] and a non-linear function is fit to these data to give the DVI. No information on the variability of salaries by draft selection number was available. As such, assume for this example that each draft selection has outcomes defined by the density of a Weibull distribution. The Weibull distribution was chosen as it is a versatile 2-parameter distribution that can be used to fit a number of types of datasets, and is a distribution commonly used in survival analysis, for which player longevity is an analogue [28]. The Weibull distribution can model high densities of near-zero DVI values at low draft picks and dome shaped value distributions at high draft picks. DVI values are typically more uncertain with increasing draft selection.

For the illustrative example, a number of DVI outcomes are simulated for particular draft selections (or combinations of draft selections), forming a distribution of player value outcomes. The Weibull distribution’s shape and scale parameters (*k*, λ) have been chosen purely for illustrative purposes. Assume the AFL DVI follows the equation

DVI(S)=−693.455ln(S)+3000, (1)

for draft selection *S* (Eq (1) was estimated from salary data in AFL (2015) and corresponds to the AFL DVI). This equation defines the mean (player value) for the Weibull distribution. Furthermore, assume the shape parameters for draft selections 1, 9 and 18 (referred to as S1, S9 and S18) are respectively *k* = 10, 3, and 1.25 (chosen to allow larger variance with draft selection). The scale parameter λ is set in order to maintain the mean derived from Eq (1) for a particular selection *S*,

$$\lambda (S,k)=DVI(S)/\Gamma (1+\frac{1}{k})$$ (2)

where Γ(.) is the gamma function. The distribution of DVI values associated with a single selection was estimated by randomly drawing 40,000 samples assuming the Weibull distribution parameterization described above. For the aggregated selections (e.g. 2 × S9) 40,000 independent random samples of the DVI values for each selection were drawn and then the paired values were summed to give the total value of the trade.

Distributional properties of DVI values resulting from single selections and the sum of DVI values due to multiple selections were compared. Specifically, we compared the mean and lower 5th percentile (risk) of draft value for selection 1 and various aggregate combinations of selections 9 and 18.

### A pragmatic example using AFL data

The previous section illustrated the concept of risk equivalence using the DVI proposed by the AFL. As no measure of uncertainty about the mean DVI was available [9] we needed to make assumptions about uncertainty. Numerous measures have been used to quantify player ability and DVI in sporting leagues, such as games played, average salary, representative games, and various on-field metrics [9, 11, 12]. For many of these measures it is possible to quantify their uncertainty. It is important to note that the focus of this section, and indeed this paper, is not the best DVI metric to use when evaluating selections, but on how once it has been chosen, it can then be used to consider mean and risk equivalency in the trading of draft selections.

The simple DVI used here (referred to as DVIg) is based upon the ratio of the number of games played by players selected in the National Draft of the Australian Football League from 1991 to 2008 relative to their draft cohort. The AFL is a closed, win-maximising league (i.e. the clubs’ primary objective is on-field success [29]) composed of 18 clubs. Details of the league and its labour market regulations can be found in Macdonald and Booth [29] and Tuck *et al*. [30]. There are three reverse-order player drafts per year: The National Draft, Pre-Season Draft and Rookie Draft, and generally all players are assigned a club via one of the drafts. The National Draft (in November) is the main player recruiting mechanism, and trading of players and draft selections can only occur during a defined exchange period prior to the National Draft. Although the example here is based upon the AFL and uses AFL data, any major league that has a draft and exchange period could utilise the concepts introduced in this paper.

While the AFL National Draft began in 1986, the initial 5 years have been removed from the calculation of DVI_g_ as talent identification was initially relatively poor (data source: AFL, 2014 or one of many online AFL statistics sites such as https://www.footywire.com/afl/footy/). Drafts after 2008 were not used (i) as rookies that are retained on club lists (rookie elevations) through selection in the draft began in 2009 (which may artificially inflate the total games played by rookie players chosen at latter draft selections), (ii) due to the influence of draft concessions allowing two expansion clubs to select quality players outside of the draft process in 2010 and 2011 (which may artificially deflate the total games played by players nominated in the initial draft selections; [30]), and (iii) due to the lower sample size of total games played (which may bias selections in one direction or another). Only players selected through the AFL National Draft were considered, and all Father-Son selections and players re-drafted were removed [30, 31]. Total games played was not used as the performance measure, as players that have not finished their career will lead to lower values for a particular draft selection than if they had completed their career.

For each draft cohort (1991 to 2008), the proportion of total games played of the cohort by each draft selection is determined (with higher draft selections likely to have played more games than lower draft selections). Namely,

$$DVIg(i,y)=\frac{\left(total\;games\;played\;for\;draft\;selection\;i\;of\;cohort\;y\right)}{\left(total\;games\;played\;for\;all\;drafted\;players\;of\;cohort\;y\right)}$$ (3)

Note that DVIg is zero when a drafted player fails to play a game (see S1 File in Supporting Information).

Statistical models were fit to the observed DVIg values using maximum-likelihood and the best-fitting model was identified using likelihood ratio tests. We chose to fit a statistical model rather than random sampling from the observed distribution of DVIg associated with each selection because the data exhibit large positive skew and there were data from only 18 annual cohorts with which to represent the distribution. The fact that many players fail to play a game means that the distribution of DVIg is best modelled as the result of two processes. First, we model the probability a player fails to play a game. Second, we model the distribution of DVIg for players, given that they have played at least one game. The probability a player selected at position *S* ≥ 1, plays no games, denoted *p*(*S*), is assumed to have the form

$$logitp(S)=\gamma_{0}+\gamma_{1}s^{\gamma_{2}},$$ (4)

where logit(*p*) = log(*p*/(1-*p*)), and the *γ*_*I*_ are constants. Eq (4) reduces to the logistic model when *γ*_2_ = 1. The expected DVIg when a player plays at least one game and is selected at position *S* is

$$\mu (S)=\frac{\alpha_{0}}{1+\alpha_{1}{\left(S-1\right)}^{\alpha_{2}}},$$ (5)

where the *α*_*i*_ are constants. Again, variation in DVIg values is assumed to follow the Weibull distribution. The shape parameter is assumed to have the form $k(S)=\beta_{0}S^{\beta_{1}}$. The scale parameter can be found using Eq (2) and replacing DVI(*S*) with *μ*(*S*). Maximum-likelihood parameters were obtained by coding the appropriate likelihood function for both models in Excel and maximising them using the Solver add-in (see S1 Table in Supporting Information). Specifically, we fitted *p*(*S*) when *γ*_2_ = 1 and when it was free, and we fitted *μ*(*S*) when *α*_2_ = 1 and when it was free. Evidence that either of these parameters differed from 1 (i.e. evidence that the simple logistic form was inadequate) was evaluated using likelihood ratio tests (LRT). We then used the best-fitting models for *p* and *μ* to simulate DVIg values and evaluated mean and risk for a number of hypothetical trades and past trades between clubs from the AFL [9].

### Trading players and draft selections

Thus far we have only considered trades of draft selections between clubs. However, it is common for trades to involve combinations of draft selections and players. It would therefore be useful to value each club’s position as part of trade negotiations regardless of whether players or selections are offered. In some respects, valuing trade options that include players should be an easier proposition, as information is known about the player’s ability and injury history, among other characteristics. This knowledge will tend to move broad probability density distributions of DVI, to much narrower distributions, which can then be aggregated in evaluating various trade combinations in the same manner as was seen for draft selections alone.

An example is provided here based upon a recent high profile trade in the AFL between the Adelaide Crows and the Geelong Cats [21]. Adelaide player Patrick Dangerfield, a marquee player in the league and the 10^th^ selection at the 2007 National Draft, requested a trade to Geelong at the conclusion of the 2015 season. Negotiations between the clubs concluded with the player and selection 50 going to Geelong, in exchange for draft selections 9, 28 and Dean Gore (an untried player and a previous selection 55 in the 2014 draft). To the end of season 2015 Dangerfield had played 154 games and was 25 years of age. Dangerfield’s individual DVIg is 0.0321 (data source: AFL, 2015), i.e. of all the players drafted in 2007, Dangerfield had played 3.21% of the total number of games played by these players to the conclusion of the 2015 season (in 2015 this ranked Dangerfield 8^th^ for players drafted in 2007; the mean DVIg across the study dataset for a selection 10 is 0.02). If we use *D* = 0.0321 as a point estimate of Dangerfield’s value in 2015, then aggregate density distributions for the Adelaide and Geelong trades can be considered. In reality, a (narrow) distribution for *D* that accounts for potential injury and changes in form, rather than a point estimate, would be preferable (his DVIg in 2019 is *D* = 0.039). In addition, the player received by Adelaide, Gore, had not played a game and is assumed to take on the full selection 55 value distribution.

## Results

### An illustrative example

**Fig 2:**
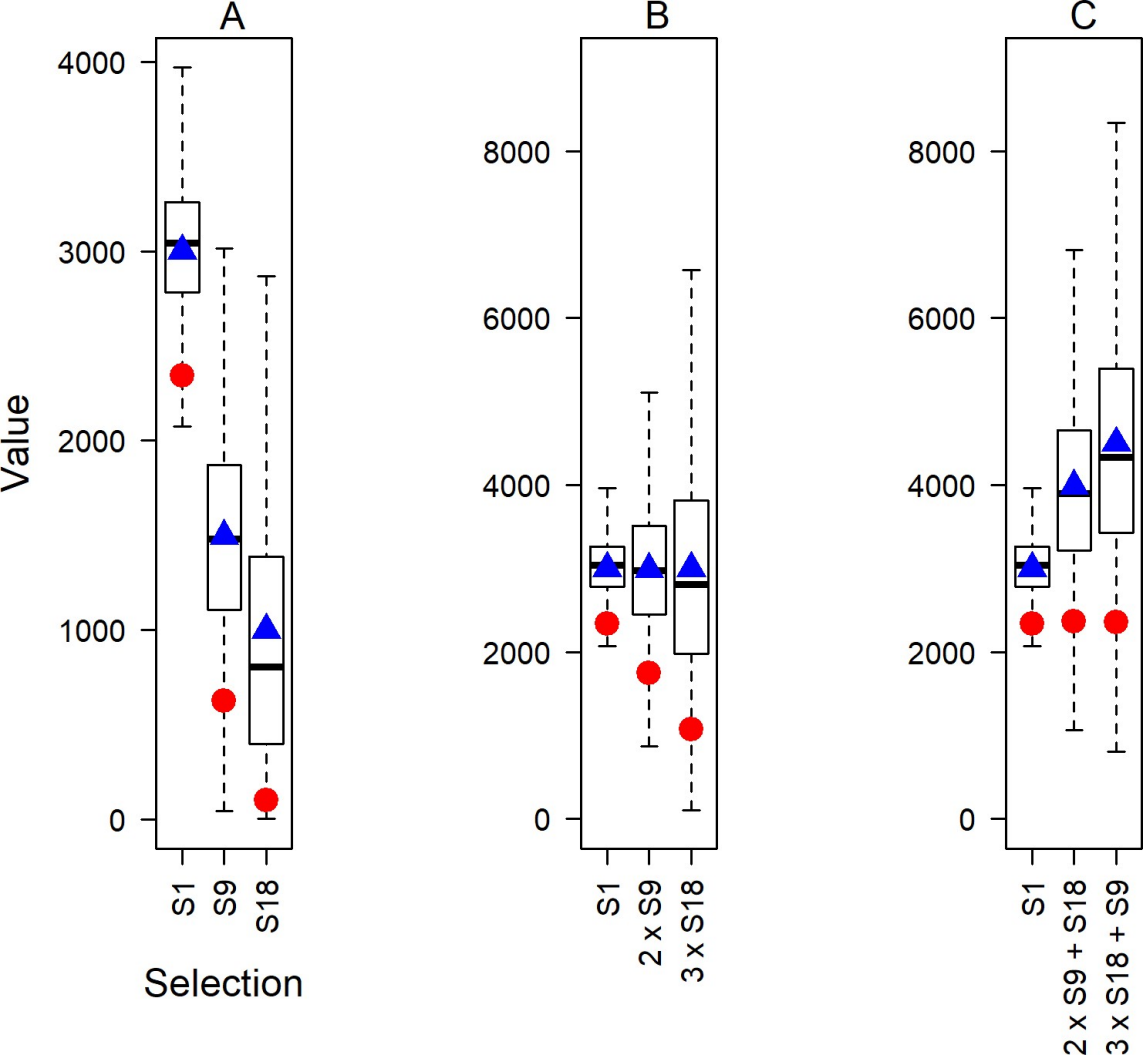
Draft value across alternative selections. Boxplots of draft value from a hypothetical example showing the mean (triangle), median (solid black line), central 50% of the distribution (box) and lower 5^th^ percentiles (circle). (A) The draft value from selections 1, 9 and 18 (S1, S9, and S18 respectively). (B) The draft value from selection 1 (S1), two of selection 9 (2 × S9), and three of selection 18 (3 × S18). (C) The draft value from selection 1 (S1), two of selection 9 and one selection 18 (2 × S9 + S18), and three of selection 18 and one selection 9 (3 × S18 + S9).

The box and whisker plot of Fig 2A illustrates the distribution of draft values for each draft selection, with decreasing value and increasing uncertainty about the draft value received as the selection number increases from selection 1 (S1) to selections 9 (S9) and 18 (S18). The mean draft values (triangle) for S1, S9 and S18 are approximately 3000, 1500 and 1000 points respectively. Fig 2B illustrates a mean value balancing strategy, whereby an equivalent mean value to S1 is sought from combinations of S9 and S18. This is achieved with two of selection 9 (2 × S9) or three of selection 18 (3 × S18). Obviously, multiple choices of a single selection are not possible, however this is allowed here (and elsewhere) for illustrative simplicity. The lower 5^th^ percentiles (red circle) are substantially less for the aggregated selections than for the single selection, S1 (Fig 2B). While the aggregated selections may provide a similar average combined value, there is increased risk that the combination of players selected is of substantially lower value (lower *MAV*) than would have been obtained from S1 alone.

In order to have equivalent risk across all strategies, one (or more) additional selections are needed for the multiple selection trade options (Fig 2C; circles). In this illustrative example, the addition of selection 18 to two selection 9s (2 × S9 + S18) or the addition of selection 9 to two selection 18s (2 × S18 + S9) leads to equivalent risk to S1. The additional selections produce an overall mean value that is greater than the single selection S1 (Fig 2C; triangles). The difference in mean values is the risk premium (also referred to as the buffer or discount factor [26]), and in this case it is equivalent to the mean value of the additional selection provided to balance risk. Risk equivalence could also have been achieved by maintaining the number of selections (two say) in order to obtain S1 but improving the draft selections on offer (better than two selections of S9).

### A pragmatic example using AFL data

**Fig 3:**
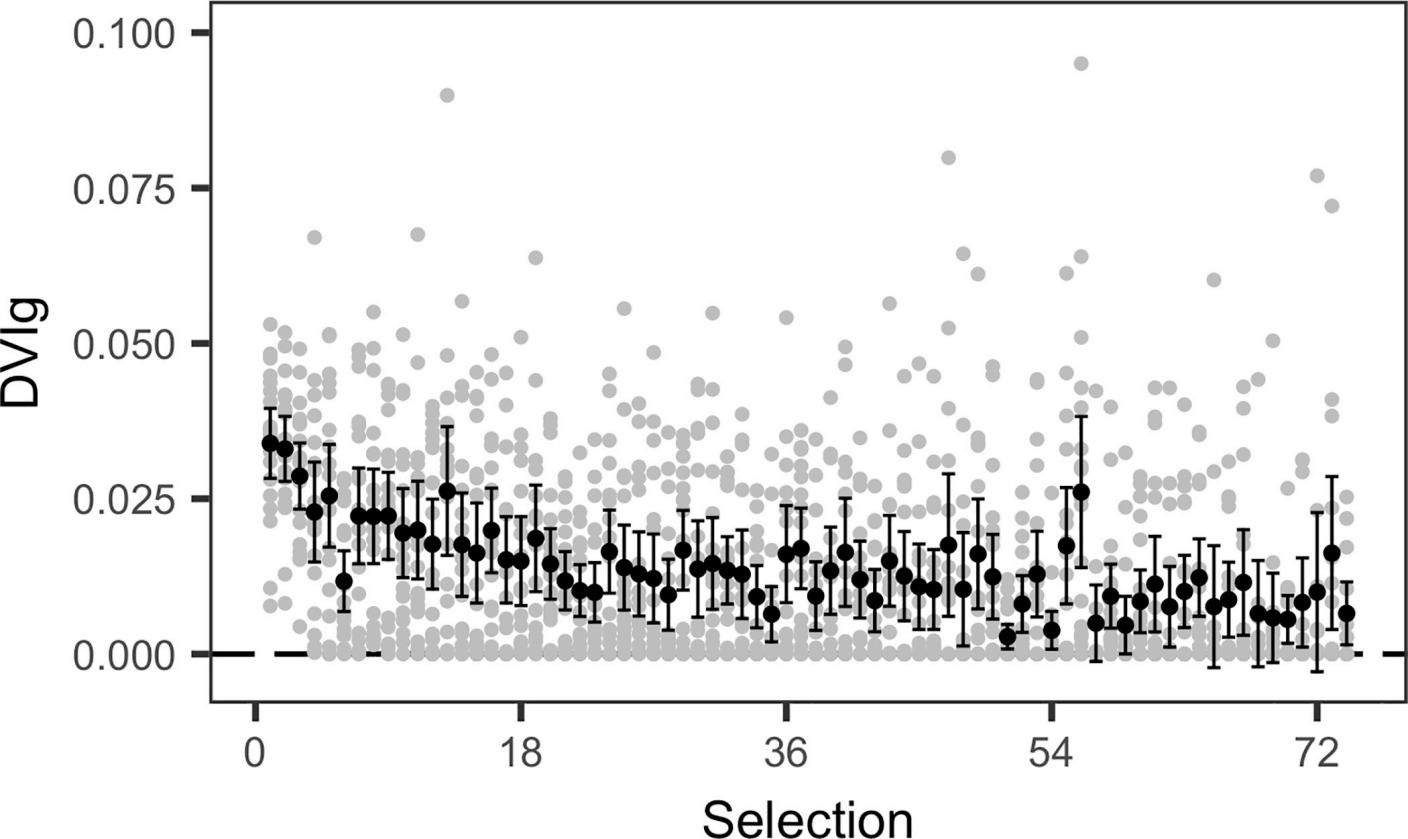
Observed DVIg values. Observed DVIg values (grey points) and the mean value at each selection (black point). Error bars depict two standard errors of the mean and approximate a 95% confidence interval for the mean.

On average, DVIg was observed to decline from high to low draft selections (Fig 3). Also evident is the increasing and large number of zero values for lower draft selections. All players drafted before selection 5 played at least one game. For the NFL, Hurley et al [11] and Schuckers [19] show figures with similar characteristics when considering games played, games started, career approximate value and Pro-Bowl appearances as a function of draft selection.

**Fig 4:**
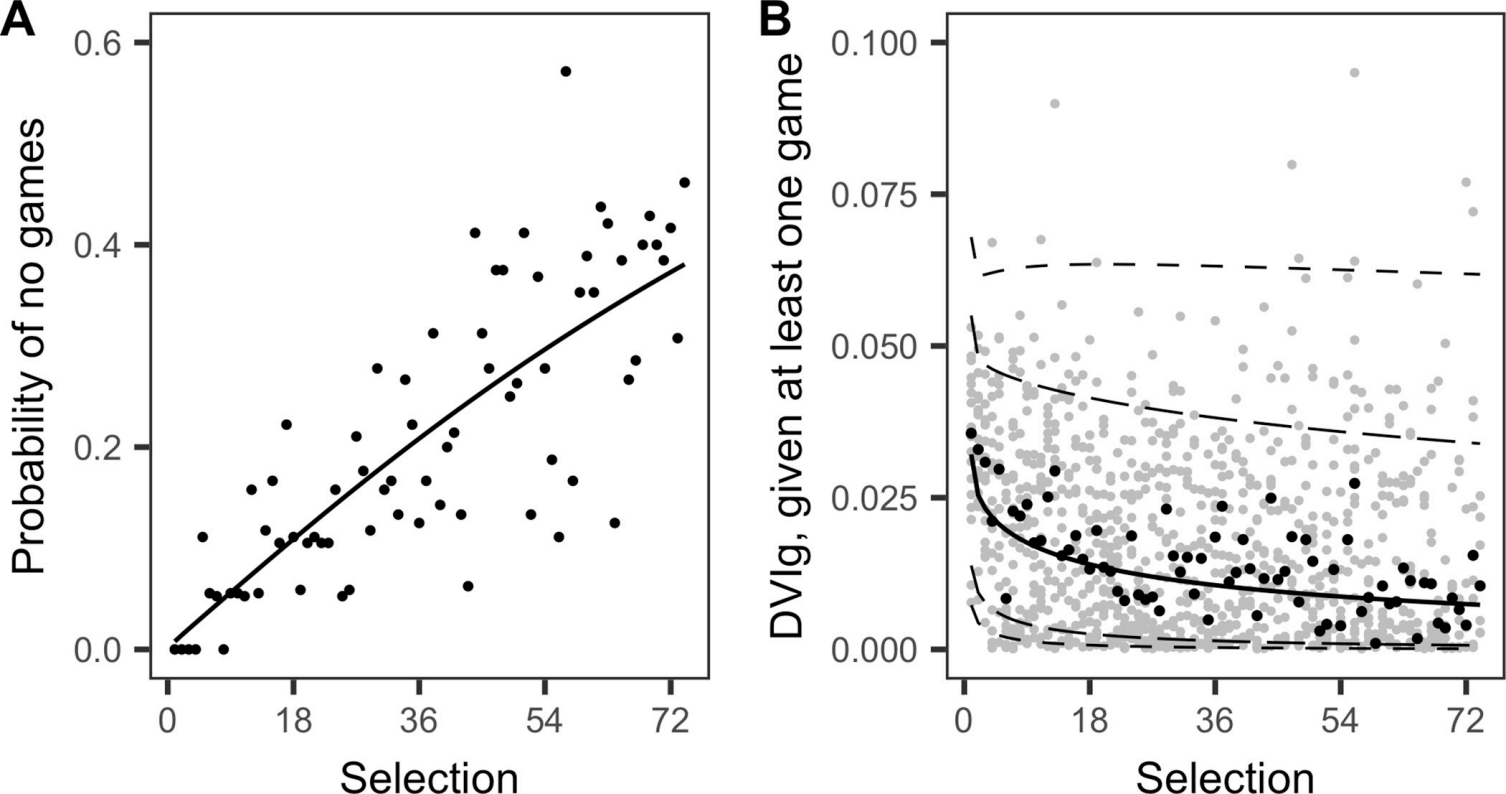
Estimated DVIg values. (A) Observed and best-fit estimate of the probability a player fails to play a game in relation to their selection. (B) Observed (grey circles) and best-fit DVIg distribution for players that played at least one game. Black circles depict medians of the observations. Variation in DVIg is modelled using the Weibull distribution. Solid line depicts the predicted median. Short- and long-dashed lines depict the bounds associated with the 95% and the 80% prediction intervals, respectively.

There was strong evidence that the probability of a player failing to play a game could be better described by Eq (4) with *γ*_2_ ≠ 1 (LRT, *G*_1_ = 8.13, *P* = 0.004). Fig 4A shows that the data is consistent with our best-fit model. The probability of failing to play a game rose steadily with selection position and players selected after 36 had a greater than 20% chance of never playing. For those players that did play at least one game, their expected DVIg could be better described when *α*_2_ ≠ 1 (LRT, *G*_1_ = 13.62, *P* < 0.001). Maximum likelihood values for the model parameters are provided in S1 Table. Fig 4B shows that the best-fit model was able to reproduce the distribution of observed DVIg values, and the expected range of values declined most during the first few selections. Even though later selected players tended to play fewer games, if players were selected after the second round (i.e. after selection 36), then their predicted distribution of DVIg values were fairly similar, irrespective of position selected (Fig 4B).

First, we consider selection 1 (S1) and risk equivalences. Table 1 shows the mean and lower 5^th^ percentile (risk) DVIg values for a number of single and multiple draft selections predicted by the best-fit model. Based on the mean, the number 1 selection is equivalent to approximately two choices of selection 18 or three choices of selection 49. However, for risk equivalence based on the 5^th^ percentile, the number 1 selection is worth approximately selection 8 and selection 9 (Table 1).

**Table 1:** Examples of risk and mean equivalence with DVIg.

Selection(s)	DVIg mean	DVIg 5^th^ percentile
**S1**	**0.0333**	**0.0093**
**S18**	0.0167	0.0000
**S49**	0.0111	0.0000
**S18 + S18**	**0.0336**	0.0038
**S49 + S49 + S49**	**0.0333**	0.0019
**S8 + S9**	0.0413	**0.0092**

Examples of multiple selections that illustrate equivalence to selection 1 in terms of mean DVIg and its 5^th^ percentile (risk). Bold values within a column highlight equivalences. SX = draft selection X.

**Fig 5:**
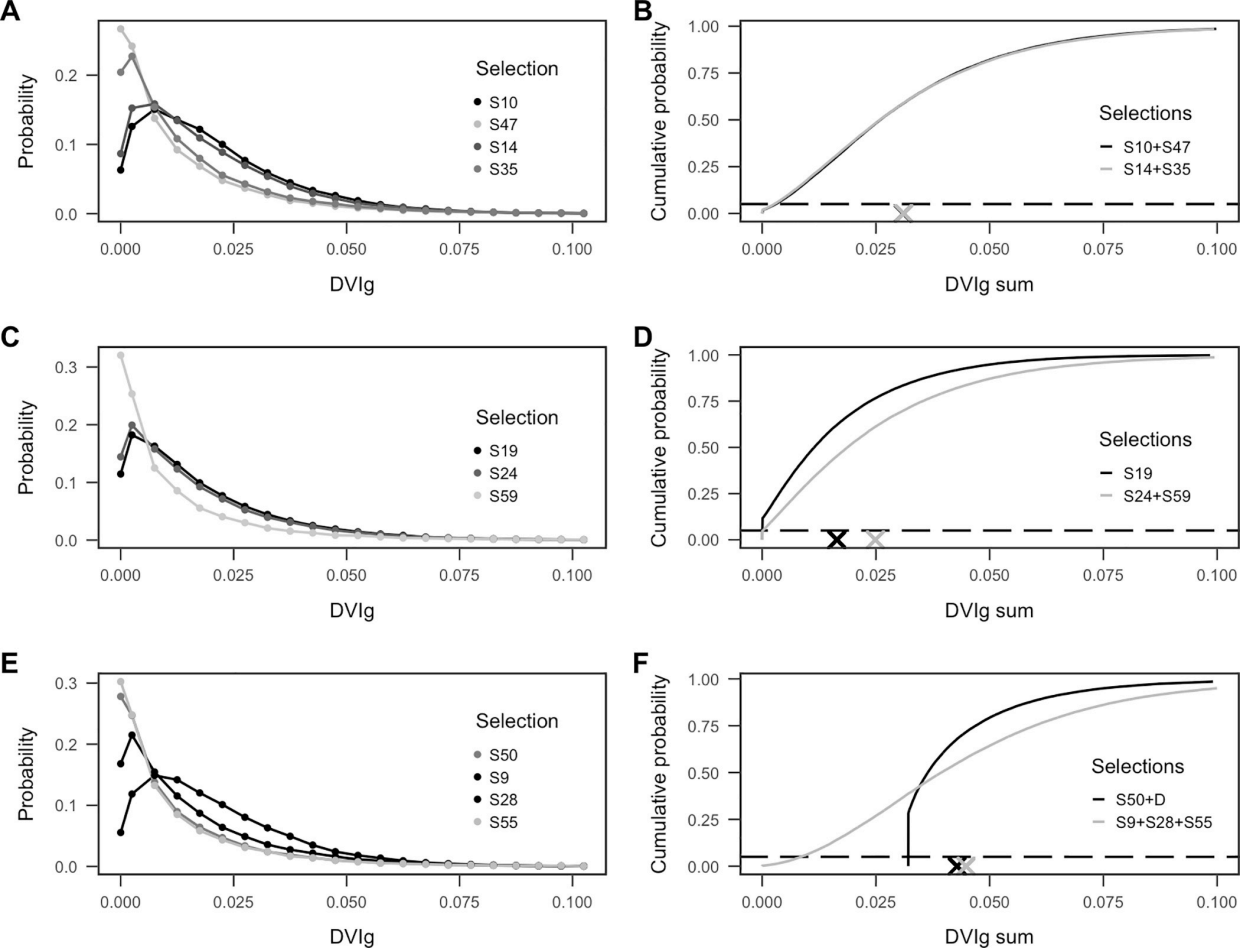
Comparison of selection distributions for AFL trades using DVIg. Probability distributions describing uncertainty in DVIg for individual selections predicted by the best-fit model (left column) and the predicted cumulative distribution of the summed DVIg when traded (right column). The probability distributions show the probability DVIg = 0 (leftmost probability) and the remaining probabilities depict the likelihood of DVIg values lying within bins of width 0.005. Dashed horizontal lines indicates the 5^th^ percentile. Crosses indicate the expected summed DVIg (i.e. mean) of the trade. A club is deemed to have benefitted if its cumulative curve is always to the right of its trading partner’s. (A-B) Adelaide and Geelong trade in 2014 [Adelaide: S10 + S47, Geelong: S14 + S35]. (C-D) Hawthorn and St. Kilda trade in 2013 [Hawthorn: S19, St. Kilda: S24 + S59]. (E-F) Geelong and Adelaide Dangerfield trade in 2015 [Geelong: D + S50, Adelaide: S9 + S28 + S55].

Past trades can also be evaluated under balance of mean value or risk metrics. The AFL [9] provide two examples of trades when considering equivalence (of means) for the DVI derived by the AFL and these two examples are illustrated in Fig 5A–5D. Remarkably, according to our model the Adelaide and Geelong trade in 2014 [Adelaide: S10 + S47, Geelong: S14 + S35] were near identical in terms of mean DVIg and risk (Fig 5A and 5B; the density curves are largely overlapping). In contrast, our model suggests that, in terms of mean and risk, the Hawthorn and St. Kilda trade in 2013 [Hawthorn: S19, St. Kilda: S24 + S59] was of benefit to St. Kilda (Fig 5C and 5D). The AFL [9] suggest that both of these examples are near equivalent in terms of mean DVI. This emphasises the importance of the DVI metric chosen.

### Trading players and draft selections

Our model predicted that for the Dangerfield trade the mean DVIg was similar for both trade combination (Fig 5E and 5F). However, the shape of the density distributions is markedly different, due to the assumed certainty of player value, *D*. As a consequence, the risk, in the form of the lower 5^th^ percentile of the distributions is markedly different. Not surprisingly, Adelaide have taken on considerably greater risk as part of this trade because the selections (and player Gore) received by Adelaide have unknown future value. For risk equivalency, Geelong would need to have provided Adelaide with additional selections, or better, proven players until the MAVs were similar (at the 5^th^ percentile level). On the other hand, a comparison of the distributions also shows that there is a possibility that the combination of value from the selections received by Adelaide will be greater than that received by Geelong. Since this trade, as a Geelong player, Dangerfield received the AFL’s Brownlow medal as the league’s best player in 2016, while Adelaide traded selection 28 for a player that has since been delisted, as has player Gore. Adelaide’s selection 9 was used on a player that remains on Adelaide’s playing list having played 43 games to the end of the 2018 season.

## Discussion

Negotiations between rival sporting clubs that wish to trade players or draft selections is difficult due to the largely unknown potential value of recruited players. Clubs are under considerable pressure to ensure trades are acceptable to their members and supporters, as the future successes of the clubs are entrusted to the skill of their list managers when selecting players in the draft and negotiating satisfactory trades. As such, an ability to estimate potential player productivity (in relative terms, if not absolute) can assist all parties in these negotiations [5, 12].

Trading players and draft selections implicitly involves risk. This risk is amplified when there is considerable probability that the player(s) received in a trade or chosen in the annual draft will not contribute to the club. Distributions of player value (or productivity) as a function of draft selection number show very high densities of low values (across a number of different performance metrics) at low draft selections (Fig 3) [11]. Even at relatively high draft selections, the chance of obtaining a player of low or zero value is substantial (from selection 5 onward for DVIg; Fig 4A). The extremely skewed distribution of DVI values implies that mean performance may not be appropriate for trade negotiations. Clubs, especially those trading high draft selections for multiple low selections (trading down), may want to ensure that the minimum acceptable value for the selection(s) held prior to the trade is maintained after the trade (Fig 2C). To achieve this, additional selections or players over and above those traded under mean equivalency may be required to balance risk, which we refer to as the risk premium. Under risk equivalency, a retrospective analysis of trades will appear to show that a club trading down receives more value than a club trading up (Fig 2C). However, whether trading up or down, risk equivalence under the proposed strategy is maintained.

In the NFL, if the Chart was conditioned on trades between 1987 and 1990 that were largely based upon clubs intuitively balancing risk, then the rapid decline seen in the Chart would be expected (Fig 1B)–more so than if the Chart were based on equivalence of expected mean performance (Fig 1A). As found by Massey and Thaler [12], recent trading of draft selections should be equivalent in terms of value from the Chart (as clubs are using this to inform their trades), but mean actual performance value would be less for a high draft selection player that was traded down when compared to the aggregate performance of the traded lower selection players. This is illustrated in Fig 2C, where mean aggregate value is greater (triangles), but risk is equivalent. If this is indeed the case for the NFL, then the Chart is not wrong, but is simply based on an alternative measure of selection value, namely risk-aversion. This conjecture is an area of future research consideration for the NFL.

Risk can be defined in many different ways [26]. In this paper, it is assumed that clubs desire a common minimum acceptable value, MAV, that has equivalent risk probability (here taken to be *p* = 0.05; there is a 5% chance of obtaining a player(s) of less value than the MAV). Of course, some club administrators may be willing to accept greater (risk-loving; *p* = 0.10 say) or less risk (risk-averse; *p* = 0.01 say) in the trade. Clubs having opposing views about levels of risk (or for that matter, the measure of central tendency) in trade negotiations could, unfortunately, lead to further protracted discussions. Having an agreed function (a DVI) to value players and draft selections, and an agreed currency (whether risk or central tendency) would alleviate some of the contention involved in trade negotiations.

In this paper, and elsewhere, it is assumed that summed value is the appropriate metric when comparing trades involving more than one selection or player [9, 11]. However, summation is problematic as can be seen from the example of trading a single 3000 point draft selection for say 30 selections averaging 100 points in value [11]. In addition, summing player values from these DVIs also does not consider value across the playing list where players may have been chosen for specific on-field roles, it does not consider the age of the players being traded, differing priorities of clubs depending on their perceived time to a championship, nor the influence of the size of the playing and team list (Hurley et al [11] does account for player type). As such, future research on the use of DVI in trading should consider alternative weighting schemes that, for example, down-weight value in proportion to the number of players or selections packaged and the number of expected career years remaining. Nonetheless, summed value is simple to apply, easily understood and is a well utilised metric, and is appropriate here in order to illustrate the concept of balancing risk.

## Conclusion

In this paper, the concept of risk equivalency in trade negotiations between sporting clubs is introduced. Examples illustrate how, once a DVI probability density surface is estimated, risk can be calculated for various combinations of draft selections and players, and comparisons can be made between trading strategies based upon balancing mean market value and balancing risk. Club list managers and recruiting staff can then ensure that packaged trades have an equivalent probability of a minimum acceptable aggregate player value. The examples provided were illustrative and future research should consider alternative DVIs appropriate to the leagues under consideration. Agreed DVI surfaces can then be utilised in the manner illustrated in this paper to facilitate negotiations and ensure risk is appropriately accounted for in trades.

## Supporting information

S1 File The DVIg data by selection number and year that underlies the model. (CSV)

S1 Table Maximum likelihood parameter estimates. (DOCX)
