# Supplementary material for: Risk equivalence as an alternative to balancing mean value when trading draft selections and players in major sporting leagues
Source: PLoS One. 2019 May 24;14(5):e0217151. doi: 10.1371/journal.pone.0217151 (PMC6534347; doi:10.1371/journal.pone.0217151)
Supplement: S1 Table — (DOCX) [file pone.0217151.s002.docx]

**Table S1**: **Maximum likelihood parameter estimates.**

| Parameter | MLE | Equation | Description |
| --- | --- | --- | --- |
| *γ*_0_ | -14.382 | *p*(*S*) | Constant term |
| *γ*_1_ | 9.541 | *p*(*S*) | Coefficient term for *S* |
| *γ*_2_ | 0.087 | *p*(*S*) | Power term for *S* |
| *α*_0_ | 0.034 | *μ*(*S*) | DVIg when *S* = 1 |
| *α*_1_ | 0.226 | *μ*(*S*) | Coefficient term for *S* |
| *α*_2_ | 0.440 | *μ*(*S*) | Power term for *S* |
| *β*_0_ | 2.234 | *k*(*S*) | Coefficient term for *S* |
| *β*_1_ | -0.243 | *k*(*S*) | Power term for *S* |

Maximum likelihood parameter estimates (MLEs) for the best AIC model fitted to the AFL DVIg data set. The associated equation and a brief description are provided for each parameter.
